# Association between Equol Production Status and Nonalcoholic Steatohepatitis

**DOI:** 10.3390/ijms222111904

**Published:** 2021-11-02

**Authors:** Takemi Akahane, Daisuke Kaya, Ryuichi Noguchi, Kosuke Kaji, Haruna Miyakawa, Yukihisa Fujinaga, Yuki Tsuji, Hiroaki Takaya, Yasuhiko Sawada, Masanori Furukawa, Koh Kitagawa, Takahiro Ozutsumi, Hideto Kawaratani, Kei Moriya, Tadashi Namisaki, Hitoshi Yoshiji

**Affiliations:** 1 Department of Gastroenterology, Nara Medical University, Kashihara 634-8521, Japan; kayad@naramed-u.ac.jp (D.K.); rnoguchi@naramed-u.ac.jp (R.N.); kajik@naramed-u.ac.jp (K.K.); fujinaga@naramed-u.ac.jp (Y.F.); tsujih@naramed-u.ac.jp (Y.T.); htky@naramed-u.ac.jp (H.T.); yasuhiko@naramed-u.ac.jp (Y.S.); furukawa@naramed-u.ac.jp (M.F.); kitagawa@naramed-u.ac.jp (K.K.); ozutaka@naramed-u.ac.jp (T.O.); kawara@naramed-u.ac.jp (H.K.); moriyak@naramed-u.ac.jp (K.M.); tadashin@naramed-u.ac.jp (T.N.); yoshijih@naramed-u.ac.jp (H.Y.); 2 Saga Nutraceuticals Research Institute, Otsuka Pharmaceutical Co., Ltd., Saga 842-0195, Japan; Miyakawa.Haruna@otsuka.jp

**Keywords:** equol, nonalcoholic steatohepatitis, estrogen, menopause

## Abstract

Equol is a metabolite of daidzein, a major soybean isoflavone with estrogenic and antioxidant activities. As the production of equol depends on the presence of certain members of the intestinal microflora, not all individuals can produce equol. We examined the relationship between NASH histological features and equol production. In an animal model, obese OLETF rats were intraperitoneally injected with a porcine serum to augment liver fibrogenesis. Equol-rich soy product, SE5-OH was orally administered during the experimental period. Treatment with SE5-OH markedly attenuated the development of liver fibrosis and expression of alpha-smooth muscle actin. In clinical research, 38 NAFLD patients (13 men and 25 women) were included. The degree of fibrosis and ballooning in equol-nonproducers was significantly higher than in equol-producers in women. The percentage of nonproducers with NAFLD activity score (NAS) ≥ 5 was significantly higher than that of producers. None of the histological features were significantly different between nonproducers and producers in men. Decision tree analysis identified predictors for NAS ≥ 5 in women. The status of equol production was the strongest predictor, followed by fasting glucose. Since equol can be noninvasively detected in urine, it can be applied as a screening tool for the progression of NASH in women.

## 1. Introduction

Nonalcoholic fatty liver disease (NAFLD) is the most common chronic liver disease globally, representing a significant health burden worldwide. Briefly, NAFLD includes a broad range of conditions, including simple steatosis and nonalcoholic steatohepatitis (NASH) [1]. The pathophysiology of NASH is multifactorial, involving genetic and epigenetic factors, insulin resistance, adipose-derived hormones, and nutritional factors [2]. The most potent driver of NASH is lipotoxicity-induced hepatocyte death, which triggers inflammation and fibrosis, leading to cirrhosis or liver cancer. 

After menopause, women have an increased risk of insulin resistance, hyperlipidemia, and visceral fat accumulation, all of which are known risk factors of NAFLD [3]. A higher incidence of NAFLD is found in postmenopausal women than in premenopausal women, as estrogen inhibits stellate cell activation and fibrogenesis, suggesting a correlation with the progression of NAFLD/NASH [4].

Soy-based foods, which are largely consumed in Asian countries, have become popular in non-Asian countries because of their beneficial effects on health. Equol, which is produced by the gut microbiota in the gastrointestinal tract, is a metabolite of daidzein, a major isoflavone found in soybean [5]. Equol has been reported to exert estrogenic activity, with an affinity for both ER alpha and ER beta estrogen receptors. Equol is superior to all other isoflavones in terms of its antioxidant activity [6]. As the production of equol depends on the presence of certain intestinal microflora, not all individuals have the ability to produce equol from the metabolism of isoflavones [7]. Only 25–30% of the adult population in Western countries can produce equol after consuming soy-based foods containing isoflavones [8,9,10]. This percentage is significantly lower than the reported 50–60% frequency of equol-producers in adults from Japan, Korea, and China [11,12,13], or Western adult vegetarians [14]. Usui et al. reported that the ratio of equol nonproducers in overweight or obese Japanese women was lower than the generally reported range [15]. Yoshikaka et al. reported that equol production was significantly associated with lower arterial stiffness and uric acid levels, and a high ratio of eicosapentaenoic acid to arachidonic acid in women in their 50s [16]. As NAFLD/NASH has been associated with both the visceral fat area and metabolic syndrome, and the incidence of NAFLD/NASH is known to increase after menopause in women, the ability to produce equol is expected to be related to the pathogenesis of NAFLD/NASH; however, to date, no relationship has been reported between them. 

Otsuka Long-Evans Tokushima fatty (OLETF) rats develop multiple metabolic and hormonal abnormalities that have similar features as obesity in humans [17,18]. OLETF rats spontaneously develop obesity, hypertriglyceridemia, hyperglycemia, hyperinsulinemia, and insulin resistance. We have previously reported the pathogenesis of NASH liver fibrosis in OLETF rats treated with porcine serum as a model for liver fibrosis in NASH under metabolic syndrome conditions [19,20]. 

Therefore, we aimed to examine the relationship between the pathogenesis of NASH and equol in animal and clinical studies.

## 2. Results

### 2.1. Animal Study

SE5-OH Ameliorated Porcine-Serum-Induced Liver Fibrosis in OLETF Rats

**Figure 1:**
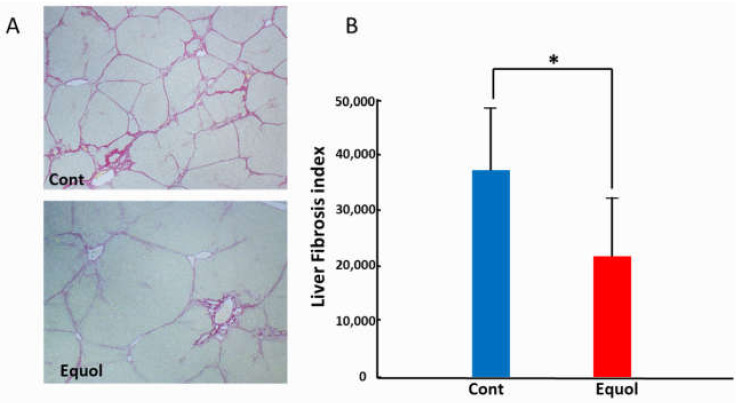
Effect of equol on the liver fibrosis development in the OLETF rats. (**A**) Representative sirius red staining photomicrographs of the liver in OLETF rats (original magnification, ×40). (**B**) Semi-quantitative analysis of the development of liver fibrosis by an image analyzer. Equol-rich soy product (SE5-OH) significantly suppressed liver fibrosis development. Data represent the mean ± SD values. * *p* < 0.01, indicating a significant difference between groups.

**Figure 2:**
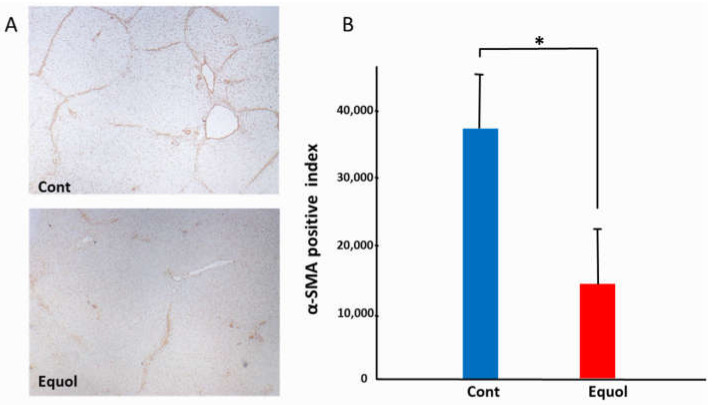
Effect of equol on the activated hepatic stellate cells (HSCs) in the OLETF rats. (**A**) Representative αSMA immunohistochemistry in OLETF rats (original magnification, ×40). (**B**) Semi-quantitative analysis of the αSMA-positive HSCs. The αSMA-positive cells were significantly suppressed by treatment with SE5-OH. Data represent the mean ± SD values. * *p* < 0.01, indicating a significant difference between groups.

Histological findings were evaluated after a 6-week treatment with porcine serum and SE5-OH. Porcine serum-induced hepatic fibrosis was ameliorated by SE5-OH treatment (Figure 1). Treatment with SE5-OH decreased αSMA-positive activated hepatic stellate cells (HSCs) (Figure 2).

### 2.2. Clinical Research

#### 2.2.1. Subject Baseline Characteristics

Although the exact amount of soy products consumed is unknown, all NAFLD participants were in the habit of consuming soy products. We found that the number of equol nonproducers was 23 out of 38. The urine equol of all nonproducers was below the limit of quantitation. The prevalence of diabetes was shown to be significantly lower in nonproducers than in producers. We did not detect any significant differences in any of the other parameters tested between the two groups (Table 1). When subjects were stratified by sex, seven male (53%) and eight female (32%) patients were identified as equol producers. No significant differences were detected in any of the tested parameters between nonproducers and producers in men. We observed, however, that in women, the prevalence of diabetes was significantly lower, whereas the prevalence of dyslipidemia was higher, although not significantly different (*p* = 0.054) in nonproducers compared to producers (Table 2). Among nonproducers, fourteen patients (82.4%) were menopausal, two had irregular menstruation and one had amenorrhea. Among the producers, seven patients (87.5%) were menopausal, and the other was 46 years old and had undergone a total hysterectomy. There was no significant difference in the frequency of menopause between producers and non-producers. The mean age at menopause was 47.6 ± 6.2 years for nonproducers and 51.4 ± 1.0 years for producers, with no significant difference between them.

#### 2.2.2. Comparison of Pathological Features between Equol Nonproducers and Producers

We compared the four histological features of NAS (steatosis, lobular inflammation, hepatocellular ballooning, and fibrosis) between nonproducers and producers stratified by sex (Table 3). We noticed that in men, none of the histological features were significantly different between nonproducers and producers. In contrast, we found that in women, the degree of fibrosis in nonproducers was significantly higher than that in producers. Although we did not observe any stage 4 fibrosis in producers, the percentage of stage 4 fibrosis in nonproducers was found to be 23.5%. Likewise, the degree of ballooning in nonproducers was shown to be significantly higher than that in producers, with the percentage of ballooning score 2 in nonproducers being 58.8%, whereas in producers the percentage was 12.5%.

**Figure 3:**
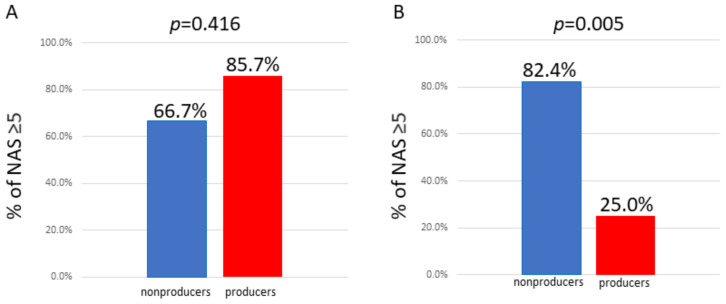
Comparison of the percentage of individuals with NAS ≥ 5 between equol nonproducers and producers. (**A**) Male; the percentage of nonproducers with NAS ≥ 5 was not significantly different from that in producers. (**B**) Female; the percentage of nonproducers with NAS ≥ 5 was significantly higher than that in producers.

We compared the percentage of nonproducers and producers with NAS ≥ 5. We accordingly found that in men, the percentage of nonproducers with NAS ≥ 5 was not significantly different from that in producers. However, in women, the percentage of nonproducers with NAS ≥ 5 was demonstrated to be significantly higher than that of producers (Figure 3).

#### 2.2.3. Prediction Model for NAS ≥ 5 Using Decision Trees Analysis

**Figure 4:**
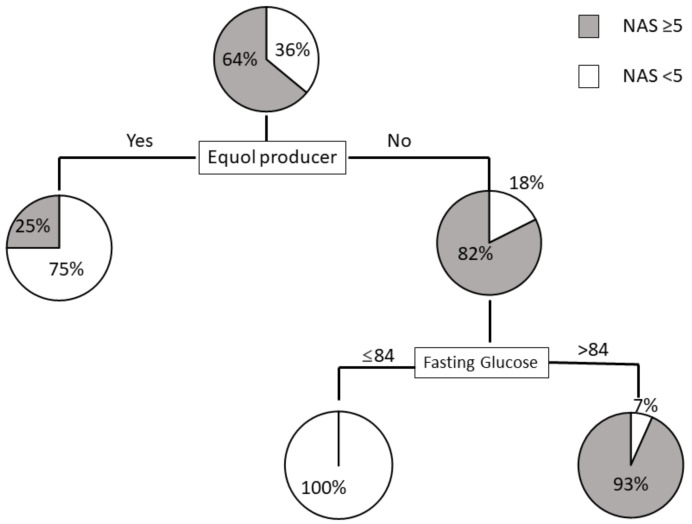
Decision tree analysis of factors predicting NAS ≥ 5 in women. Pie graphs indicate the proportions of patients with NAS ≥ 5 and NAS < 5.

To clarify the profiles associated with NAS ≥ 5, we created a decision-tree algorithm. We set the following factors as independent variables: age, menopause, hypertension, dyslipidemia, diabetes mellitus, BMI, platelet count, fasting glucose, HOMA-IR, ferritin, type 4 collagen 7S, P-III-P, FIB-4, APRI, and equol producers. Among the factors expected to predict NAS ≥ 5, we selected equol producers as the initial distinguishable factor for NAS ≥ 5 in women. We observed a NAS ≥ 5 in 25% of equol producers. In contrast, a NAS ≥ 5 was identified in 82% of equol nonproducers. Fasting glucose was demonstrated to be the second most distinguishable factor among equol nonproducers. We observed a NAS ≥ 5 in 93% of nonproducers with fasting glucose levels >84 mg/dL. In contrast, none of the nonproducer patients with fasting glucose ≤ 84 mg/dL was found to have a NAS ≥ 5 (Figure 4). The mean fasting glucose levels were not significantly different between equol nonproducers with NAS ≥ 5 (103.1 ± 15.4 mg/dL) and those with NAS < 5 (88.0 ± 16.6 mg/dL) (*p* = 0.143).

## 3. Discussion

In this study, we showed that treatment with SE5-OH markedly attenuated the development of liver fibrosis and the expression of alpha-smooth muscle actin in male OLETF rats, and in the clinical study, the degree of fibrosis and ballooning in equol nonproducers was significantly higher than that of producers in women with NASH. In addition, the percentage of nonproducers with NAS ≥ 5 was significantly higher than that of producers in women with NASH. In contrast, in men with NASH, no difference was identified between nonproducers and producers. As most women in this study were menopausal and the menstruation of nonproducers who had not reached menopause was irregular or amenorrheic, it could be suggested that the status of equol production is associated with the pathogenesis of NASH in menopausal women or women with low estrogen secretion. 

The percentage of equol producers in Asia has been reported to be approximately 50–60% [12,21]. In this study, 53% of men with NASH were equol producers, similar to a previous report [21]. However, in women with NASH, the percentage of equol producers was 32%, lower than previously reported [12] and similar to the percentage of equol producers in obese women reported by Usui et al. [15]. Equol is produced from the isoflavone daidzein in the gut of humans and animals through the action of certain bacterial biotypes [22]. Gut dysbiosis has been significantly associated with obesity and the development and progression of NAFLD. The progression from NAFLD to NASH is known to largely occur due to bacterial dysbiosis, which activates inflammatory and profibrogenic intracellular pathways via Toll-like receptors and the activation of the inflammasome [23]. Bacterial dysbiosis might also be one of the causes of the inability of the host to produce equol, which in turn might be one of the reasons for the progression of NASH.

In women, the proportion of those with dyslipidemia was higher among equol nonproducers than in producers, although the difference was not significant (*p* = 0.054). Some studies have reported a relationship between the level of serum lipids and the ability to produce equol. Yoshitaka et al. reported that equol producers had significantly lower visceral fat area, as well as lower levels of triglycerides and high-density lipoprotein (HDL) cholesterol than did nonproducers [16]. Likewise, Guo et al. reported that women equol producers had significantly lower levels of low-density lipoprotein (LDL) cholesterol than did nonproducers [24]. Estrogen is known to increase the levels of HDL cholesterol and decrease those of LDL cholesterol, influencing body fat deposition. In addition, equol has been reported to exert the strongest estrogenic activity among all known isoflavones or isoflavone-derived metabolites [25,26]. Both the central adiposity and levels of serum LDL cholesterol are known to increase in menopausal women. Therefore, the production of equol has been suggested to be associated with postmenopausal central adiposity and lipid metabolism. 

Epidemiological studies have indicated that the increased intake of dietary soy isoflavones positively correlates with a lower incidence of diabetes and increased tissue sensitivity to insulin [27,28,29]. However, evidence on the association between soybean intake and type 2 diabetes is inconsistent [30,31,32]. OLETF rats exhibit visceral fat accumulation obesity due to overeating. Plasma triglycerides are elevated at six weeks of age, plasma cholesterol is elevated at 21 weeks of age, blood glucose is elevated at 18 weeks of age, and most individuals are diagnosed as diabetic at 25 weeks of age. In the animal study, we demonstrated that equol inhibited the progression of NASH in the obese rats that did not develop diabetes because of the administration of pig serum and equol twice weekly for six weeks to 8-week-old OLETF rats. In our clinical study, the proportion of women with diabetes mellitus was higher in producers than in nonproducers. Therefore, in women’s non-producers, NASH may develop and progress before the onset of diabetes. 

The diagnosis of NASH is established by the presence of a characteristic pattern of steatosis, inflammation, and hepatocellular ballooning on liver biopsies. Accordingly, a NAS threshold value (NAS ≥ 5) has been used for the diagnosis of NASH [33,34]. Our decision-tree analysis results showed that in women, the ability of equol production was the strongest predictor of NAS ≥ 5, with fasting glucose being the second strongest predictor of NAS ≥5 in equol nonproducers. As equol is thought to affect lipid metabolism and oxidative stress, a combination of the inability to produce equol and glucose metabolism abnormalities might increase the risk of NASH progression. As equol can be noninvasively measured in urine samples, it could be used as a useful screening tool for the prediction of NASH in women.

Equol is known to be superior to all other isoflavones in its antioxidant and estrogenic activities. Moreover, equol has been reported to be effective in improving the ischemic cardiovascular risk profile [35], menopausal symptoms [36,37], and suppression of decreased bone mineral density [38,39]. We used male OLETF rats to avoid the effects of estrogen in female rats. This can be considered a model to test the effect of equol administration in NASH. As SE5-OH markedly attenuated the development of liver fibrosis, equol intake as a therapeutic strategy might improve NASH. We administered 2000 mg/kg/day of SE5-OH (13 mg/kg/day of equol), which has been reported as a no-observed-adverse-effect level for developmental effects [40]. It has been reported that the serum level of equol was 14 ng/mL in male Sprague–Dawley rats given a single dose of 2000 mg/kg/day SE5-OH [41]. On the other hand, the Cmax of equol in plasma was reported to be 1.15 ± 1.31 ng/m after a single dose of 10 mg equol was administered to 12 men and women aged 45−65 years [42]. The median intake of daidzein was reported to be 12.1 mg/day from a 1-day dietary survey of 1232 participants, and 9.5 mg/day from a 16-day dietary survey of 88 participants, where both surveys were conducted in Japanese [43]. Although the exact amount of equol converted from daidzein cannot be estimated because of the individual differences in equol production ability, the amount of equol administered to rats was much higher than the amount of equol obtained from diets in humans. Although equol has an ameliorating effect on liver fibrosis in male OLETF rats, the ability to produce equol was not associated with the progression of NASH in men. This may be due to differences in equol concentrations. The status of equol production in both men and women has not changed over the years. However, due to estrogen depletion after menopause in women, the antioxidant and estrogenic activity of equol may have a suppressive effect in the progression of NASH. The optimal amount of equol that is safe but sufficient to inhibit the progression of NASH needs to be investigated in future research.

Our study had limitations. First, the sample size was small. Second, as this was an observational study, we could not make any conclusion on the causal relationship, and the cross-sectional design did not identify whether equol nonproducers progressed to NASH. Third, this was a single-center study. Forth, although all of the subjects were in the habit of consuming soy products, the exact amount of soy products consumed was unknown. Fifth, an imaging tool such as transient elastography is clinically useful for identifying advanced fibrosis in patients with NAFLD. However, we did not evaluate liver fibrosis by imaging tools such as transient elastography. 

In conclusion, equol production status is closely related to the progression of NASH in women. Equol intake as a therapeutic strategy might lead to the improvement of NASH.

## 4. Material and Methods

### 4.1. Animal Study

#### 4.1.1. Animal Treatment

Male OLETF rats were supplied by Otsuka Pharmaceutical Co. (Tokushima, Japan) [17]. Equol-rich soy product (SE5-OH) was supplied by Otsuka Pharmaceutical Co. At the age of eight weeks, the OLETF rats were divided into two groups. Each group consisted of six rats. Porcine serum (0.5 mL) was intraperitoneally injected in all rats twice a week for six weeks. Through drinking water, one group was treated with equol-rich soy product, SE5-OH (2000 mg/kg/day) which contained 0.65% equol, during the experimental period [40]. At the end of the experiment, the rats were anesthetized, their abdominal cavities were opened, and their livers were harvested for histological evaluation. All animal procedures were performed according to a standard protocol and in accordance with the NIH Guide for the Care and Use of Laboratory Animals. All experiments were approved by the Animal Care and Use Committee of Nara Medical University.

#### 4.1.2. Immunohistochemical Staining and Semi-Quantification

The liver sections were routinely stained with sirius red to detect the development of liver fibrosis. Immunohistochemical staining of a-smooth muscle actin (α-SMA) (Dako, Kyoto, Japan) was performed as described previously [44,45]. Semi-quantitative analyses were conducted using ImageJ software (National Institutes of Health, Bethesda, MD, USA).

### 4.2. Clinical Research

#### 4.2.1. Study Populations

Forty Japanese patients with clinically suspected NASH (13 men and 27 women) who visited the Department of Gastroenterology, Nara Medical University Hospital (Kashihara, Japan) between October 2017 and March 2019, were recruited for this study. All patients underwent a liver biopsy. The upper limit of alcohol consumption for NASH was defined as less than 30 g/d for men and less than 20 g/d for women in terms of ethanol content. Exclusion criteria included positivity for hepatitis B surface antigen or anti-hepatitis C virus antibodies, other hepatobiliary diseases, antibiotics or hormone therapy, diarrhea, and inability to consume soy food products due to allergy or preference. Following the exclusion of two women who did not consume soy food products due to preference, a total of 38 (13 men and 25 women) patients were enrolled in this study. 

#### 4.2.2. Clinical and Laboratory Assessments

Patient information was collected using a questionnaire that included questions on alcohol consumption, medication history, and soy food consumption habits. In accordance with the definition of the Japanese Society of Obstetrics and Gynecology, women without a menstrual period for >12 months were considered menopausal. Hypertension was defined as a systolic blood pressure ≥140 mmHg, diastolic blood pressure ≥90 mmHg, or current use of antihypertensive medication. Diabetes mellitus was defined as a fasting blood glucose level ≥126 mg/dL or current use of anti-diabetic medication. Dyslipidemia was defined as the current use of lipid-lowering medication. Insulin resistance was assessed using the homeostasis model assessment-insulin resistance (HOMA-IR), which was calculated as fasting insulin (μU/mL) × fasting glucose (mg/dL)/405. The FIB-4 index for noninvasive markers of liver fibrosis was calculated as follows:

(1) $$\mathrm{FIB}4\;\mathrm{index}\;=\left(\mathrm{age}\;\left[y\right]\times \;\mathrm{AST}\right)/\left(\mathrm{platelet}\;\mathrm{count}\;\left[10^{9}/L\right]\times \surd \mathrm{ALT}\right)$$

The aspartate aminotransferase to platelet ratio index (APRI) for noninvasive markers of liver fibrosis was calculated as follows:

(2) $$\mathrm{APRI}=[\mathrm{AST}\;\left(\mathrm{IU}/l\right)/\mathrm{AST}\;\left(\mathrm{upper}\;\mathrm{limit}\;\mathrm{of}\;\mathrm{normal}\;\mathrm{IU}/L\right)/(\mathrm{platelet}\;\mathrm{count}\;\left[10^{9}/L\right]\times 100$$

#### 4.2.3. Analysis of Urinary Isoflavone and Equol

To determine the equal-producing status, participants consumed soy food products containing approximately 50 mg isoflavones twice a day, and urine samples were collected the following morning. The levels of daidzein and equol in the urine of patients were measured in the Saga Nutraceuticals Research Institute (Otsuka Pharmaceutical Co. Ltd.) by high-performance liquid chromatography (HPLC; Nexera X2; Shimadzu, Japan) using a type C18 column (Cortecs C18, 2.7 μm, 3.0 × 150 mm; Waters, Milford, MA, USA) and an SPD-M30A PDA and RF-20Axs detection system (Shimadzu), according to a modified method by Lundh et al. [46]. This method has been used for the detection of metabolites in human urine in previous studies [47]. Briefly, 100 μL urine sample was deconjugated by incubating with 2 μL (ca. 300 U) β-glucuronidase (G0876, Sigma-Aldrich, St. Louis, MO, USA) in sodium acetate buffer (pH 5.0) at 37 °C for 30 min. Samples were then extracted using OASIS HLB microelution plates (Waters) before HPLC analysis. Quantitation was performed using UV response (254 nm and 280 nm) for daidzein and fluorescence response (Ex: 255 nm, Em: 310 nm) for equol. The laboratory precision was assessed for each batch of samples through the analysis of the standard solution. The limits of quantification (LOQ) for daidzein and equol were 0.076 and 0.080 nmol/mL, respectively. The equol-producing status of each urine collection was defined by using a log-transformed equol/daidzein ratio of −1.75 or more [14].

### 4.3. Pathology

The liver tissue biopsy specimens were stained with hematoxylin-eosin, silver, and Azan stain, and analyzed by experienced pathologists blinded to the clinical data. NASH was defined as fat accumulation in more than 5% of hepatocytes, inflammation, and hepatocellular ballooning on liver biopsies. Disease activity was scored using the NAFLD activity score (NAS) of the Nonalcoholic Steatohepatitis Clinical Research Network. The scoring system comprised four histological features: steatosis (0–3), lobular inflammation (0–2), hepatocellular ballooning (0–2), and fibrosis (0–4) [33,34]. Accordingly, NAS is the unweighted sum of steatosis, lobular inflammation, and hepatocellular ballooning scores. The substages (1a, 1b, and 1c) of fibrosis stage 1 were combined as a single stage.

### 4.4. Statistical Analysis

All data were expressed as the mean ± SD. Continuous variables were compared between the two groups using the Student’s *t*-test or the Mann–Whitney *U* test, whereas categorical variables were compared using the Chi-squared test. Statistical significance was set at *p* < 0.05. A decision-tree algorithm was constructed to reveal the profiles associated with a NAS ≥ 5. The following factors were set as independent variables: age, menopause, hypertension, dyslipidemia, diabetes mellitus, BMI, platelet count, fasting glucose, HOMA-IR, ferritin, type 4 collagen 7S, P-III-P, FIB-4, APRI, and equol producers. All calculations were performed using SPSS software (version 27, IBM Corp., Armonk, NY, USA).

### 4.5. Ethics Approval

This study was approved by the Ethics Committee of Nara Medical University (approval no. 1607). The study was conducted in concordance with the Helsinki Declaration. All included patients signed an informed consent form before they participated in the study. All research was performed in accordance with relevant guidelines and regulations.

## Figures and Tables

**Table 1:** Clinical characteristics of equol nonproducers and producers.

Variable	Nonproducer (*n* = 23)	Producer (*n* = 15)	*p*-Value
Age (y)	54.6 ± 15.0	60.4 ± 9.3	0.697
Sex (Male/Female)	(6/17)	(7/8)	0.191
Equol (nmol/mL)	BLQ	23.0 ± 34.2	
Daidzein (nmol/mL)	50.2 ± 46.1	12.0 ± 7.4	0.01
Log (Equol/Daidzein)	-	0.0 ± 0.75	
Hypertension (%)	11 (47.8)	7 (46.7)	0.944
Dyslipidemia (%)	11 (47.8)	5 (33.3)	0.376
Diabetes Mellitus (%)	11 (47.8)	12 (80.0)	0.047
BMI (kg/m^2^)	28.6 ± 4.2	28.5 ± 4.1	0.945
Platelet count (×10^4^/μL)	20.3 ± 6.4	16.4 ± 5.5	0.062
AST (IU/L)	62.0 ± 26.7	62.3 ± 35.4	0.97
ALT (IU/L)	78.8 ± 45.4	73.0 ± 41.2	0.696
Fasting glucose (mg/dL)	103.6 ± 24.7	115.7 ± 30.0	0.205
HOMA-IR	5.8 ± 5.8	11.6 ± 11.1	0.129
Ferritin	173.5 ± 192.9	238.0 ± 128.0	0.278
Type 4 collagen 7S (ng/mL)	6.2 ± 3.1	6.0 ± 2.2	0.822
P-III-P (U/mL)	0.7 ± 0.3	0.7 ± 0.2	0.895
FIB-4 index	2.5 ± 1.6	3.3 ± 2.7	0.258
APRI	1.1 ± 0.7	1.5 ± 1.3	0.214

Values are *n* (%) or mean ± standard deviation. BMI, body mass index; AST, aspartate aminotransferase; ALT, alanine aminotransferase; HOMA-IR, homeostasis model assessment-insulin resistance, type III procollagen peptide; P-III-P, the aspartate aminotransferase to platelet ratio index; APRI, below the limit of quantitation; BLQ.

**Table 2:** Comparison of clinical characteristics between equol nonproducers and producers stratified by sex.

	Male			Female		
Variable	Nonproducer (*n* = 6)	Producer (*n* = 7)	*p*-Value	Nonproducer (*n* = 17)	Producer (*n* = 8)	*p*-Value
Age, y	53.4 ± 17.9	56.0 ± 8.4	0.734	60.5 ± 14.0	64.1± 8.8	0.512
Equol (nmol/mL)	BLQ	34.3 ± 46.5		BLQ	11.7 ± 8.9	
Daidzein (nmol/mL)	52.1 ± 41.7	10.7 ± 7.9	0.059	49.5 ± 48.9	13.4 ± 7.1	0.011
Log (Equol/Daidzein)	-	0.17 ± 0.73		-	−0.2 ± 0.8	
Hypertension (%)	3 (50.0)	4 (57.1)	0.797	8 (47.1)	3 (37.5)	0.653
Dyslipidemia (%)	2 (33.3)	4 (57.1)	0.391	9 (52.9)	1 (12.5)	0.054
Diabetes Mellitus (%)	4 (66.7)	5 (71.4)	0.853	7 (41.2)	7 (87.5)	0.03
Menopause	-	-	-	14 (82.4)	7 (87.5)	0.743
BMI (kg/m^2^)	28.9 ± 2.6	28.5 ± 3.7	0.852	28.5 ± 4.7	28.5 ± 4.7	0.997
Platelet count (×10^4^/μL)	19.3 ± 4.9	17.6 ± 5.0	0.552	20.6 ± 6.9	15.4 ± 6.0	0.078
AST (IU/L)	60.2 ± 16.3	60.0 ± 24.3	0.989	62.6 ± 30.1	64.4 ± 44.7	0.907
ALT (IU/L)	94.5 ± 45.7	78.4 ± 45.6	0.54	73.2 ± 45.3	68.3 ± 39.4	0.797
Fasting glucose (mg/dL)	113.3 ± 40.7	129.9 ± 27.8	0.405	100.1 ± 16.5	103.3 ± 27.6	0.725
HOMA-IR	9.4 ± 9.8	16.7 ± 14.7	0.397	4.4 ± 2.9	8.6 ± 8.2	0.101
Ferritin	273.8 ± 352.4	242.6 ± 140.0	0.834	144.0 ± 117.2	233.4 ± 126.5	0.111
Type 4 collagen 7S (ng/ML)	4.8 ± 0.9	5.6 ± 2.9	0.634	6.5 ± 3.3	6.3 ± 1.7	0.853
P-III-P (U/mL)	0.8 ± 0.3	0.7 ± 0.2	0.831	0.7 ± 0.3	0.7 ± 0.2	0.803
FIB-4 index	1.9 ± 1.0	2.5 ± 1.1	0.414	2.7 ± 1.8	4.1 ± 3.5	0.317
APRI	1.1 ± 0.3	1.3 ± 0.6	0.495	1.1 ± 0.7	1.7 ± 1.7	0.358

Values are *n* (%) or mean ± standard deviation. BMI, body mass index; AST, aspartate aminotransferase; ALT, alanine aminotransferase; HOMA-IR, homeostasis model assessment-insulin resistance, type III procollagen peptide; P-III-P, the aspartate aminotransferase to platelet ratio index; APRI, below the limit of quantitation; BLQ.

**Table 3:** Comparison of pathological features between equol nonproducers and producers stratified by sex.

	Male			Female		
	Nonproducer *n*, (%)	Producer *n*, (%)	*p*-Value	Nonproducer *n*, (%)	Producer *n*, (%)	*p*-Value
Fibrosis stage			0.292			0.047
0	1, (16.7)	0, (0)		2, (11.8)	0, (0)	
1	1, (16.7)	0, (0)		3, (17.6)	0, (0)	
2	1, (16.7)	1, (14.3)		4, (23.5)	7, (87.5)	
3	3, (50.0)	3, (42.9)		4, (23.5)	1, (12.5)	
4	0, (0)	3, (42.9)		4, (23.5)	0, (0)	
Steatosis			0.629			0.44
1	0, (0)	1, (14.3)		6, (35.3)	5, (62.5)	
2	5, (83.3)	5, (71.4)		7, (41.2)	2, (25.0)	
3	1, (16.7)	1, (14.3)		4, (23.5)	1, (12.5)	
Lobular inflammation			0.489			0.262
0	0, (0)	0, (0)		1, (5.9)	0, (0)	
1	2, (33.3)	2, (28.6)		5, (29.4)	5, (62.5)	
2	3, (50.0)	5, (71.4)		11, (64.7)	3, (37.5)	
3	1, (16.7)	0, (0)		0, (0)	0, (0)	
Ballooning			0.612			0.03
1	5, (83.3)	5, (71.4)		7, (41.2)	7, (87.5)	
2	1, (16.7)	2, (28.6)		10, (58.8)	1, (12.5)	
NAS score			0.292			0.084
3	0, (0)	1, (14.3)		2, (11.8)	3, (37.5)	
4	2, (33.3)	0, (0)		1, (5.9)	3, (37.5)	
5	2, (33.3)	4, (57.1)		9, (52.9)	1, (12.5)	
6	1, (16.7)	2, (28.6)		4, (23.5)	1, (12.5)	
7	1, (16.7)	0, (0)		1, (5.9)	0, (0)	

## Data Availability

The datasets generated during the current study are available from the corresponding author on reasonable request.
